# Applications of Immunomagnetic Reduction Technology as a Biosensor in Therapeutic Evaluation of Chinese Herbal Medicine in Tauopathy Alleviation of an AD *Drosophila* Model

**DOI:** 10.3390/bios12100883

**Published:** 2022-10-17

**Authors:** Ming-Tsan Su, Chen-Wen Lu, Wen-Jhen Wu, Yong-Sin Jheng, Shieh-Yueh Yang, Wu-Chang Chuang, Ming-Chung Lee, Chung-Hsin Wu

**Affiliations:** 1 School of Life Science, National Taiwan Normal University, Taipei 11677, Taiwan; 2 MagQu Co., Ltd., Taipei 11231, Taiwan; 3 Sun Ten Pharmaceutical Co., Ltd., New Taipei City 231632, Taiwan; 4 Brion Research Institute of Taiwan, New Taipei City 231632, Taiwan

**Keywords:** Alzheimer’s disease, tauopathy, immunomagnetic reduction, traditional herbal medicine, *Drosophila melanogaster*

## Abstract

Alzheimer’s disease (AD) is the most common form of dementia. The most convincing biomarkers in the blood for AD are currently β-amyloid (Aβ) and Tau protein because amyloid plaques and neurofibrillary tangles are pathological hallmarks in the brains of patients with AD. The development of assay technologies in diagnosing early-stage AD is very important. The study of human AD subjects is hindered by ethical and technical limitations. Thus, many studies have therefore turned to AD animal models, such as *Drosophila melanogaster*, to explore AD pathology. However, AD biomarkers such as Aβ and p-Tau protein in *Drosophila* *melanogaster* occur at extremely low levels and are difficult to detect precisely. In this study, we applied the immunomagnetic reduction (IMR) technology of nanoparticles for the detection of p-Tau expressions in hTau^R406W^ flies, an AD *Drosophila* model. Furthermore, we used IMR technology as a biosensor in the therapeutic evaluation of Chinese herbal medicines in hTau^R406W^ flies with Tau-induced toxicity. To uncover the pathogenic pathway and identify therapeutic interventions of Chinese herbal medicines in Tau-induced toxicity, we modeled tauopathy in the notum of hTau^R406W^ flies. Our IMR data showed that the selected Chinese herbal medicines can significantly reduce p-Tau expressions in hTau^R406W^ flies. Using evidence of notal bristle quantification and Western blotting analysis, we confirmed the validity of the IMR data. Thus, we suggest that IMR can serve as a new tool for measuring tauopathy and therapeutic evaluation of Chinese herbal medicine in an AD *Drosophila* model.

## 1. Introduction

With the advent of the aging era, researchers predict dramatic increases in the incidence of Alzheimer’s disease (AD). Therefore, developing effective disease-modifying therapeutic interventions for AD is an urgent priority for academia and pharmaceutical companies. AD is a neurodegenerative disorder characterized by senile plaques and neurofibrillary tangles (NFTs) in the brain. Although the available therapies for AD may delay cognitive decline, they do not alter the underlying disease processes. The most convincing biomarkers in the blood for AD are currently β-amyloid (Aβ) and tau protein because amyloid plaques and neurofibrillary tangles are pathological hallmarks in the brains of patients with AD [1]. So far, the treatment or relief of AD is mainly based on western medicine. However, Chinese herbal medicines can also be used as a new choice for AD treatment or relief. One of the reasons is that Chinese herbal medicine not only has good curative effects but also has fewer side effects. In order to achieve the above purpose, our laboratory provides Chinese herbal medicine compositions for the treatment of AD. Our previous results showed that Chinese herbal medicine compositions have a great potential effect in protecting AD [2,3]. By using digital holographic microscopy, we evaluated the therapeutic effect of Chinese herbal medicines in curing neurodegeneration through detecting soma volume and neurite outgrowth of living AD neurons [4]. However, to the best of our knowledge, until now, these assay technologies have been tools used only for research. There is a lack of validation for the therapeutic evaluation of Chinese herbal medicine via AD biomarker detection by using any of these assay technologies.

Human genetics studies have improved the understanding of AD-related genes. However, the study of human subjects is hindered by ethical and technical limitations. Many studies have therefore turned to AD animal models, such as *Drosophila melanogaster*, to explore AD pathology [5]. Targeted expression of Aβ and Tau proteins in adult flies can lead to changes in structural appearance, including loss of an eye, and loss of notal bristles [6]. The amyloid hypothesis has led to extensive efforts being expended to screen medicines that can reduce brain Aβ accumulation by, for example, preventing Aβ aggregation [7]. By detecting Aβ_42_ in the brain tissue, we showed that the Chinese herbal medicine, Yi-Gan-San, can alleviate Aβ_42_ expression in an AD model of *Drosophila melanogaster* [8]. In addition to Aβ protein accumulation, the deposition of Tau, which is the second pathological hallmark of AD, is a concern. A combination of therapies that can protect against Aβ and Tau may be a key therapeutic strategy for AD. Tau plays a normal role in regulating microtubules and thus axonal transport in neurons [9]. However, misfolded and aggregated Tau is toxic to cells. The accumulation of neurofibrillary tangles (NFTs) induced by hyperphosphorylated Tau is the main cause and a common pathological hallmark of tauopathy. Because the presence of NFTs is highly correlated with the progression, duration, and severity of neurodegeneration, research on its pathogenesis has focused on the molecular and cellular events that lead to the abnormal formation and accumulation of NFTs [10]. The neurodegeneration in AD seems to be correlated with some form of Tau toxin. A number of studies have revealed that the density and distribution of the neurofibrillary tangles correspond to the severity of AD. The intracellular neurofibrillary tangles consist of paired helical filaments that are built out of the aberrantly misfolded and hyperphosphorylated microtubule-associated Tau protein. However, whether Chinese herbal medicines also have a function as therapeutics for alleviating tauopathy is still unclear.

**Figure 1:**
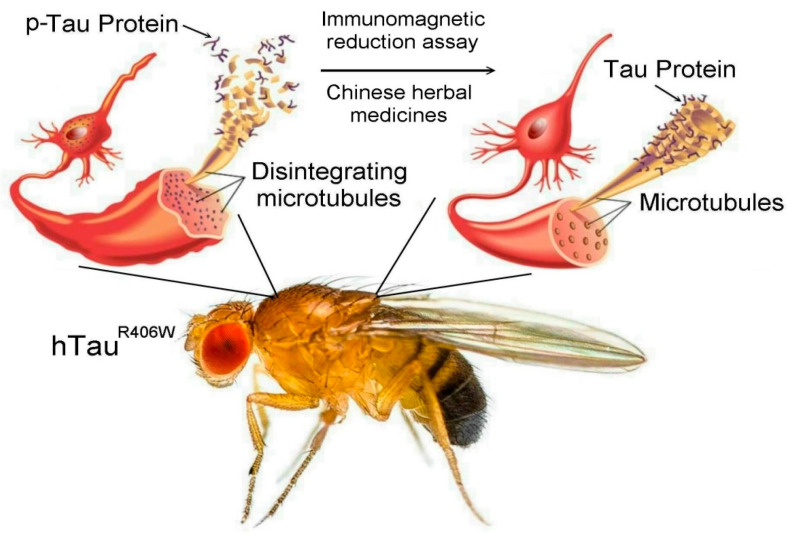
Schematic illustrating the more favorable therapeutic effects of Chinese herbal medicine treatment, which alleviates Tau-induced toxicity in hTau^R406W^ *Drosophila* flies.

Alternative AD treatment approaches target the microtubule-binding protein Tau, a component of NFTs. To uncover the pathogenic pathway and identify traditional herbal medicine therapeutic interventions in an AD model of *Drosophila melanogaster*, we modeled tauopathy in the notum of *Drosophila* and used an immunomagnetic reduction (IMR) assay. We have previously demonstrated that notum is more sensitive and more favorably suited for analyzing Tau-induced toxicity [6]. In view of AD biomarkers such as Aβ and p-Tau protein in *Drosophila* *melanogaster*, which occur at extremely low levels and are difficult to detect precisely, IMR was applied in this study, which has been successfully developed to detect extremely small amounts of p-Tau protein in the cerebrospinal fluid of human [11,12,13,14]. In this study, the preparation of reagents containing antibody-functionalized magnetic nanoparticles for assaying p-Tau protein was described. In addition, the design of the high-Tc SQUID-based IMR analyzer was also illustrated. Moreover, the characterizations of detecting p-Tau protein using the reagents and the analyzer were explored. Hundreds of AD *Drosophila melanogaster* with Tau-induced toxicity were analyzed with IMR measurements for p-Tau protein to investigate the feasibility of the therapeutic evaluation of Chinese herbal medicine in alleviating tauopathy (Figure 1).

## 2. Materials and Methods

### 2.1. Chinese Herbal Medicine Preparation

The Chinese herbal medicine of Yi-Gan-San in this study was prepared according to the protocol suggested by Sun-Ten Pharmaceutical Company; regarding the mass fraction, the Yi-Gan-San constituents were Bupleurum 2 g, licorice 2 g, chuanxiong 3.2 g, *Angelica* 4 g, *Atractylodes* 4 g, Poria 4 g, and Uncaria 4 g per 100 g of the final product. In this study, hTau *Drosophila* pupae were cultured in standard cornmeal–yeast–agar medium at 29 °C and 60% humidity. The standard medium of hTau^R406W^ *Drosophila* pupae with Yi-Gan-San treatment was evenly mixed with 1% Yi-Gan-San by weight of the medium. Considering that Yi-Gan-San must be able to dissolve sufficiently and uniformly in the medium, we chose 1% Yi-Gan-San as the highest dose to treat flies.

### 2.2. Preparation of AD hTau^R406W^ Flies with Tau-Induced Toxicity 

UAS-hTau^R406W^ transgenic flies were provided by Feany [15]. Eq-gal4 flies were used as wild-type (WT) controls. The UAS-gal4 system was used to overexpress hTau^R406W^, and a tauopathy fly model was developed by recombining UAS-hTau^R406W^ and Eq-gal4 lines (Eq > hTau^R406W^). Unless otherwise specified, all fly lines, including hTau^R406W^, Eq-gal4, and Eq > hTau^R406W^ flies, were cultured and maintained on a standard cornmeal–yeast–agar medium at 25 °C and 60% humidity with a 12:12 h on/off light cycle. 

### 2.3. IMR Assay of p-Tau Expressions in AD hTau^R406W^ Flies

In this study, we used a fourth-generation superconducting-quantum-interference-device (SQUID, MagQu, Taipei, Taiwan) instrument with 36 detection channels. In addition to characterizing the performances of SQUID-IMR instrument, the consistency in assaying proteins associated with neurodegenerative diseases among instruments was investigated. A SQUID-based AC magnetic susceptometer (XacPro-S, MagQu, Taipei, Taiwan) was used to determine time-dependent AC magnetic susceptibility, which was used to approximate the association between the magnetic nanoparticles and target protein molecules [12]. The IMR signal, which denotes the reduction in magnetic susceptibility caused by the association between magnetic nanoparticles and the target protein molecule, was detected using the magnetic susceptometer and represented the concentration of the target protein. For each sample, IMR signal measurements were performed in duplicate. Convert IMR signals to biomarker concentrations via a standard curve.

hTau^R406W^ transgenic flies, an AD *Drosophila* model with Tau-induced toxicity, and their WT (100 flies per group) were separately homogenized and then centrifuged at 2500× *g* for 15 min within 1 h of sample drawing. Tissue clarifier was filled into cryovials and stored at −20 °C. To measure the IMR signal, the sample (40–60 µL) was mixed with 80–60 µL of p-Tau detecting reagent at room temperature to detect the IMR signal (%). The p-Tau detecting reagent contained magnetic nanoparticles functionalized with monoclonal antibodies against the target protein, and the homogenate was then dispersed in phosphate-buffered saline with a pH of 7.2 (MagQu) at room temperature. The magnetic nanoparticles were dextran-coated Fe_3_O_4_ particles (MF-DEX-0060, MagQu). IMR signal measurements were performed in duplicate for each sample at each target protein concentration. The signals were converted into biomarker concentrations using standard curves. All samples were blinded for IMR measurements. The p-Tau reagent (MF-TAU-0060, MagQu) contained magnetic nano-particles immobilized with a monoclonal antibody (T9450, Sigma-Aldrich Co., St. Louis, MO, USA) against human p-Tau protein. These reagents were superparamagnetic, with a saturated magnetization of 0.3 emu/g. 

### 2.4. Bristle Quantification in AD hTau^R406W^ Flies

hTau^R406W^ transgenic flies have been used to assess the tauopathy-induced toxicity by quantifying their bristle number [5]. Over-expressions of hTau^R406W^ caused missing mechano-sensory bristles. The nota of 1-day-old adult flies were dissected in phosphate-buffered saline with 0.1% Triton X-100. Serial focal plane images of notum bristles were obtained using a stereo microscope (Leica MZFLIII, Leica Microsystems, Wetzlar, Germany) fitted with a digital camera (CoolSnap 5.0, Photometrics, Tucson, AZ, USA). Images were analyzed and bristles were scored using Photoshop software (Adobe, CA, USA).

### 2.5. Western Blotting in AD hTau^R406W^ Flies

Total proteins were extracted from head tissue of *Drosophila* flies following treatment (100 flies per group). The removed tissue was homogenized in a buffer solution on ice for 1 h and was centrifuged at 4 °C for 13,000 rpm for 20 min. The supernatant was quantified using a bicinchoninic acid protein assay kit (Thermo Fisher Scientific, Waltham, MA, USA). The proteins were separated on 12.5% or 15% sodium dodecyl sulfate polyacrylamide gels (Bionovas Pharmaceuticals, Washington, DC, USA) and were transferred to polyvinylidene difluoride membranes (GE Healthcare Life Sciences, Barrington, IL, USA). The primary antibodies were anti-Histone H3.3B (Thermo Fisher Scientific) anti-pTau 181 and anti-Tau (Cell Signaling Technology, Danvers, MA, USA) antibodies. The secondary antibodies were horseradish peroxidase (HRP)-conjugated secondary antibody (Santa Cruz Biotechnology, Dallas, TX, USA), and protein immunoreactive bands were visualized using the enhanced chemiluminescence (ECL) substrate (Millipore, Billerica, MA, USA). Band intensities were quantified using Image J analysis software (version 1.48t, Wayne Rasband, NIH, Washington, DC, USA).

### 2.6. Statistical Analysis

All data are presented as means ± standard errors of the mean. We used Kruskal–Wallis non-parametric test for multiple comparisons and followed by the Mann–Whitney non-parametric test for comparisons of two independent samples. A *p* value of <0.05 was considered significant. All data were obtained in at least three independent experiments.

## 3. Results

### 3.1. p-Tau 181 Concentration-Dependent Biomarker IMR Signals 

**Figure 2:**
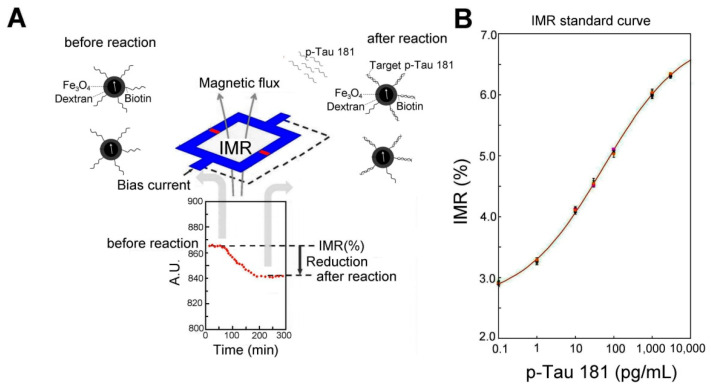
IMR detecting p-Tau 181 and standard curve: (**A**) A scheme of the physical mechanism of IMR assay. IMR is a method of assaying target molecules through measuring reductions in the mixed-frequency magnetic susceptibility of magnetic reagents resulting from the association between magnetic nanoparticles and target molecules. A magnetic nanoparticle coated with p-Tau 181 bioprobes is presented in the diagram. (**B**) Standard curve of p-Tau181 measured by IMR technique. The IMR value increased with the p-Tau 181 concentration.

Figure 2A showed the physical mechanism of the IMR assay. IMR is a method of assaying target molecules through measuring reductions in the mixed-frequency magnetic susceptibility of magnetic reagents resulting from the association between magnetic nanoparticles and target molecules. We used a reagent containing magnetic nanoparticles functionalized with monoclonal antibodies against the p-Tau protein to detect the p-Tau 181 protein. In order to specifically bind magnetic nanoparticles to specific molecules to be tested, it is often necessary to coat the surface of magnetic nanoparticles with a layer of biological probes such as the p-Tau181 antibody. The oxidized glucan is oxidized, and then the oxidized glucan is covalently bonded to the amino group in the antibody. Finally, magnetic separation technology is used to remove excess biological probes to obtain purified magnetic reagents. In order to make IMR have ultra-high sensitivity, we have developed a set of high-Tc SQUID as a magnetic sensor for magnetic immunoassay. The superconducting magnetic immunoassay consists of two parts: a sample unit and a signal-sensing unit. The sample to be tested is placed in the sample unit of the analyzer, and the SQUID is placed in the sensing unit. We used the flux transfer technology to transmit the magnetic decrement signal of the sample to be tested in the sample unit to the SQUID of the sensing unit. The magnetic nanoparticles in the magnetic fluid are thermally disturbed by water molecules, and the fluid has no spontaneous magnetic dipole in the absence of an external magnetic field. However, when a magnetic field is applied to the fluid, the magnetic moments of the magnetic particles in the liquid tend to follow the direction of the applied magnetic field, thus creating magnetization. When the applied field is removed, the magnetic nanoparticles again exhibit a zero magnetic dipole due to the thermal perturbation of water molecules. This phenomenon is called superparamagnetism. Once the antibody-functionalized magnetic nanoparticles associate with the target molecules such as p-Tau181, the hydrodynamic volume of the associated nanoparticles increases. Meanwhile, the AC magnetic susceptibility of the reagent was decreased.

A superconducting magnetic immunoassay was used to measure the relationship between different p-Tau181 concentrations and IMR signals, and the results are shown in Figure 2B. The detection sensitivity is approximately 0.1 pg/mL. The results revealed that the IMR signal increased as the p-Tau 181 concentration increased.

### 3.2. IMR in Therapeutic Evaluation of Chinese Herbal Medicine for Tauopathy Alleviation in AD hTau^R406W^ Flies 

**Figure 3:**
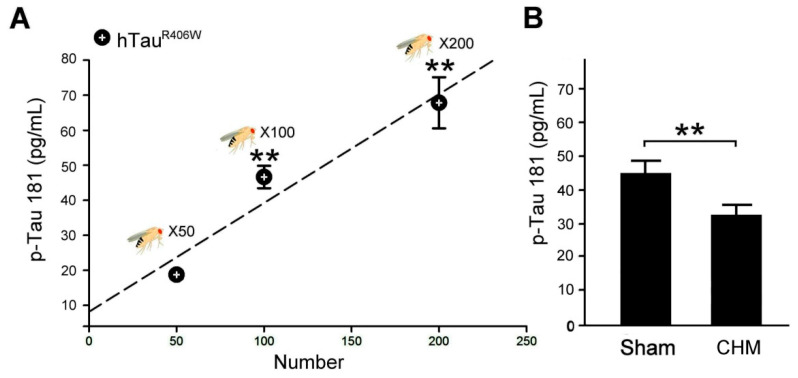
IMR can be a biosensor in therapeutic evaluation of Chinese herbal medicine for tauopathy alleviation in AD hTau^R406W^ flies: (**A**) Relationship between the p-Tau 181 concentrations and the number of hTau^R406W^ flies (sampled number = 50, 100, 200) obtained through IMR assay. (**B**) The diagram Quantified comparison of p-Tau 181 concentrations and IMR values in hTau^R406W^ flies (sampled number = 100) under sham and Chinese herbal medicine (CHM) treatment (three replicate experiments for each group). Values are mean ± SEM (** *p* < 0.01, Kruskal–Wallis non-parametric test for multiple comparisons and followed by the Mann–Whitney non-parametric test for comparisons of two independent samples).

We established the curve of the relationship between p-Tau 181 expression and the number of hTau^R406W^ flies by using an IMR assay (Figure 3A). The results revealed that the IMR signal increased as the p-Tau 181 concentration increased, which was linked to the number of hTau^R406W^ flies. IMR signals (%) for p-Tau 181 concentrations between 0.1 and 10,000 pg/mL were used to explore the analytical relationship, which followed a logistic function. Using the relationship curve presented in Figure 3A, we quantified and compared the p-Tau 181 concentration of a hundred hTau^R406W^ flies with that of the flies with sham treatment (Figure 3B). By IMR assay, the detected concentration of the p-Tau 181 protein in a hundred hTau^R406W^ flies with sham treatment was 44.7 ± 3.7 pg/mL, and 32.3 ± 2.9 pg/mL for a hundred hTau^R406W^ flies with Chinese herbal medicine treatment, the results clearly observed that hTau^R406W^ flies with Chinese herbal medicine treatment had decreased p-Tau protein. By test analysis, the *p*-value of p-Tau protein concentration to distinguish hTau^R406W^ flies with Chinese herbal medicine treatment from hTau^R406W^ flies with sham treatment was less than 0.01, revealing a significant difference in p-Tau protein concentration between hTau^R406W^ flies with Chinese herbal medicine and sham treatments. The IMR results revealed that hTau^R406W^ flies with Chinese herbal medicine treatment had more favorable therapeutic effects for tauopathy alleviation.

### 3.3. Verification of IMR Results by the Notal Bristles Quantification

**Figure 4:**
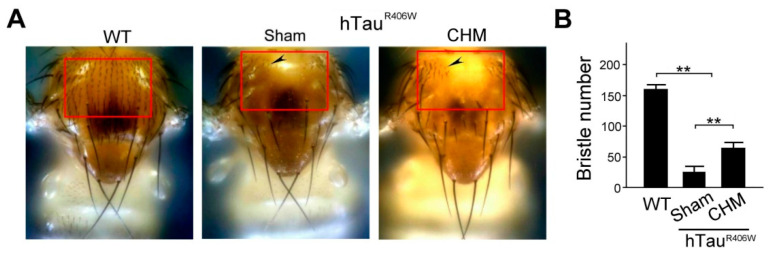
Chinese herbal medicine treatment showed favorable therapeutic effects for tauopathy alleviation by notal bristle quantification: (**A**) Numbers of notal bristle (as indicated by arrows) within the red frames of the hTau^R406W^ flies with sham and Chinese herbal medicine (CHM) treatments were notably lower than in their WT counterparts; hTau^R406W^ flies under CHM treatments demonstrated higher notal bristle numbers than the hTau^R406W^ flies under sham treatment. (**B**) Quantified comparison of the number of notal bristles among hTau^R406W^ flies under sham and CHM treatments, and their WT counterparts (sampled number of flies = 30 for each group, three replicate experiments for each group). Values are mean ± SEM (** *p* < 0.01, two-way ANOVA followed by Mann–Whitney non-parametric test for comparisons).

For further verification, we used the notal bristle quantification to assay Tau-induced toxicity in AD Drosophila melanogaster. The *Drosophila* notum-based system has been employed to assay Tau-induced toxicity in our previous study [6]. As shown in Figure 4A, the number of notal bristles in hTau^R406W^ flies was much lower than that in WT flies. The number of notal bristles was higher in hTau^R406W^ flies treated with Chinese herbal medicine treatment than in hTau^R406W^ flies with sham treatment. We quantified and compared the number of notal bristles in hTau^R406W^ flies and WT flies undergoing sham and Chinese herbal medicine treatments. The number of notal bristles of WT flies was significantly higher than that of hTau^R406W^ flies with sham and Chinese herbal medicine treatments (*p* < 0.01, Figure 4B). The number of notal bristles in hTau^R406W^ flies with Chinese herbal medicine treatments was significantly higher than that of hTau^R406W^ flies with sham treatment (*p* < 0.01, Figure 4B). The results revealed that hTau^R406W^ flies with Chinese herbal medicine treatment had more favorable therapeutic effects for tauopathy alleviation by notal bristle quantification.

### 3.4. Verification of IMR Results by the Western Blotting Assay

**Figure 5:**
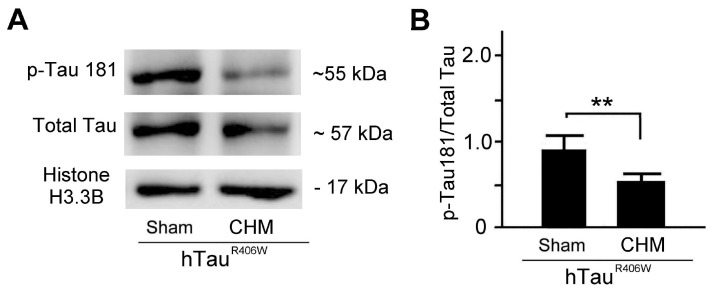
Chinese herbal medicine treatment showed favorable therapeutic effects for tauopathy alleviation by Western blotting: (**A**) Example of Western blotting analysis of p-Tau 181 and total Tau expressions in hTau^R406W^ flies and their WT counterparts under sham and Chinese herbal medicine (CHM) treatments. (**B**) Quantified comparison of the ratio of p-Tau 181 to total Tau expressions among hTau^R406W^ flies under sham and CHM treatment (three replicate experiments for each group). Values are mean ± SEM (** *p* < 0.01, Kruskal–Wallis non-parametric test for multiple comparisons and followed by the Mann–Whitney non-parametric test for comparisons of two independent samples). kDa, kilodaltons.

We further used Western blotting to analyze the ratio of p-Tau 181 to total Tau expressions in hTau^R406W^ flies under sham and Chinese herbal medicine treatments (Figure 5). The results revealed that expressions of p-Tau 181 to total Tau in hTau^R406W^ flies under sham treatment were higher than those in hTau^R406W^ flies under Chinese herbal medicine treatment (Figure 5A). We quantified and compared the ratio of p-Tau 181 to total Tau expressions in hTau^R406W^ flies under sham and Chinese herbal medicine treatments. We found that the ratio of p-Tau 181 to total Tau expressions of hTau^R406W^ flies under sham treatment was significantly higher than that of hTau^R406W^ flies under Chinese herbal medicine treatment (*p* < 0.01, Figure 5B). The results revealed that hTau^R406W^ flies with Chinese herbal medicine treatment had more favorable therapeutic effects for tauopathy alleviation by the Western blotting assay. 

## 4. Discussion

With the completion of the human and *Drosophila* genome sequences, simpler gene families in *Drosophila* have simplified many loss-of-function studies that have given us a fundamental understanding of disease-related genes. The smaller gene family of *Drosophila* is also a potential advantage for drug discovery, as fewer genes need to be regulated to establish sensitive conditions for drug screening. A good example is given by *Drosophila* notal bristle as a novel assessment tool for the pathogenic study of Tau toxicity and the screening of therapeutic compounds [6]. As a multicellular organism, *Drosophila* allows the modeling of complex traits relevant to humans. Many complex traits share genetic determinants in flies and humans, so we can reasonably expect that what is found in flies will be applicable to humans, and many complex disease phenotypes that are applicable to *Drosophila* models are also applicable to drug screening. Unlike neurodegenerative diseases, normal neuronal function relies on interactions between multiple cell types, for example, between neurons and dendrites, between neurons and muscles, and between neurons and glia. Because Chinese herbal medicines that correct abnormal neuronal function need to function in a multi-cellular environment, it makes sense to screen them in vivo models.

This study used *Drosophila melanogaster* as a good model for screening Chinese herbal medicines. The modeling of human tauopathy in Drosophila results in the manifestation of relevant phenotypes, hTau *Drosophila melanogaster* is similar to human and mammalian models of tauopathy [16]. The accumulation of insoluble Tau aggregates in the form of typical neurotoxic neurofibrillary tangles triggers the pathogenesis of tauopathy in *Drosophila melanogaster*. Therefore, the hTau *Drosophila melanogaster* has become a very important tool for in-depth analysis of neurofibrillary tangles in neurodegeneration and for screening drugs that are useful to inhibit the toxicity of Tau aggregates. However, AD biomarkers of p-Tau protein in hTau *Drosophila* *melanogaster* occur at extremely low levels and are difficult to detect precisely. Thus, we applied the IMR technology of nanoparticles for the detection of p-Tau expressions in hTau^R406W^ flies, an AD *Drosophila melanogaster* model. To achieve ultra-high sensitivity, a high-Tc supercon SQUID ac magnetosusceptometer was designed and applied to detect the tiny reduction in the ac magnetic susceptibility of the reagent. Using the reagent and this analyzer, extremely low concentrations of the p-Tau 181 protein in human plasma could be detected. Further, the feasibility of identifying subjects in early-stage AD via assaying the p-Tau 181 protein is demonstrated. Their results show a diagnostic accuracy for prodromal AD higher than 80% and reveal the possibility of screening for early-stage AD using SQUID-based IMR [14]. As shown in Figure 1A, the SQUID element used in the superconducting magnetic immunoanalyzer is basically composed of two superconducting Josephson junctions in parallel. Under an appropriate bias current, the SQUID can convert a very weak magnetic signal into an electronic circuit—the measured voltage signal. SQUID is currently the most sensitive detector to magnetic signals, and its noise is only about one-thousandth of the magnetic signal of magnetic nanoparticles. Therefore, even a very small number of magnetic nanoparticles in the reagent, when combined with the biomolecules to be tested, cause only a very small change in the alternating magnetic quantity. This very small amount of AC magnetic change can also be detected by the SQUID. Therefore, using SQUID for magnetic decrement measurements should have a very high sensitivity to p-Tau181. As far as we know, our study is the first application of IMR technology to detect p-Tau and Tau protein expressions in the *Drosophila* tissue for the diagnosis of AD.

To verify the validity of the IMR data, we further used notal bristle quantification and Western blotting analysis to double-check that the selected Chinese herbal medicines can significantly reduce p-Tau expressions. As shown in Figure 4, we quantified the number of notal bristles to assay Tau-induced toxicity in AD *Drosophila melanogaster*. To elucidate the Tau gain-of-toxicity functional mechanism and to identify potential treatments, we overexpressed human Tau variants (hTau) in the dorsal mesothorax (notum) of *Drosophila*. The overexpression of Tau variants caused a loss of notal bristles, and the phenotype was used to evaluate the toxicity of ectopic p-Tau. The notum of *Drosophila melanogaster* may thus serve as a new tool for measuring Tau-induced toxicity, and it can assist in screening new drugs for possible therapeutic interventions. The bristle-loss phenotype was highly associated with the toxicity of p-Tau in flies. We demonstrated that the bristle-loss phenotype could be rescued by Chinese herbal medicines treatment. Additionally, decreasing the endogenous Tau dosage was beneficial because it ameliorated the bristle-loss phenotype. The bristle-loss phenotype was used to evaluate the efficacy of potential therapeutic compounds. In this study, a 36-channel instrument utilizing IMR was used. To achieve ultra-high sensitivity, a SQUID AC magnetometer was designed and applied to detect the tiny decrease in the AC magnetic susceptibility of the reagent. We compared assay results from three independent instruments. The channel-to-channel variation in measured biomarker concentrations ranged from 2.09% to 5.62%. The assay accuracy was found to range from 99% to 103.7%. Across the three instruments, *p*-values were higher than 0.05 in the measured concentrations of any tested biomarkers. Our results show that the SQUID-IMR instrument has high throughput, high stability, and high consistency [17]. Currently, a 36-channel, SQUID-based, high-Tc AC magnetometer can detect very low concentrations of amyloid and Tau protein in human plasma. In this study, we used the sensor to detect very low concentrations of tau protein in *Drosophila melanogaster*. Many laboratory animal experiments also used the sensor to detect very low concentrations of amyloid and tau protein in mice and rats [18]. According to our ongoing experiments, we used the sensor to detect neurofilament light chain (NfL) in dog plasma. NfL is a neuron-specific cytoskeletal protein expressed in axons. Damaged axons of the central nervous system release NfLs into the cerebrospinal fluid (CSF) and the blood. In humans and dogs with neurologic diseases, NfL is also used as a biomarker.

By Western blotting analysis, our results showed that Chinese herbal medicines can significantly reduce the ratio of p-Tau 181/total Tau protein in the hTau *Drosophila melanogaster* (Figure 5). It is generally accepted that elevated t-tau and p-tau levels at least partially reflect the degree of neuronal damage in AD. It has been reported that patterns of cerebral glucose metabolism typical of AD are significantly related to elevated p-tau181 levels but not to elevated p-tau levels [19]. Thus, we examined the ratio of p-Tau 181/total Tau protein in the hTau *Drosophila melanogaster*. Tauopathy represents a group of neurodegenerative disorders that are characterized by insoluble intraneuronal and glial fibrillar lesions known as neurofibrillary tangles. Tau is a neuron-specific microtubule-binding protein, which is required for the integrity and functioning of neuronal cells, and the hyperphosphorylation of Tau and its subsequent aggregation, resulting in the major pathogenic mechanisms of tauopathy in human and mammalian model systems. By IMR technology of nanoparticles for the detection of p-Tau 181 expressions, our results revealed that hTau^R406W^ flies with Chinese herbal medicine treatment had therapeutic effects for tauopathy alleviation. 

## 5. Conclusions

To uncover the pathogenic pathway and identify therapeutic interventions of Chinese herbal medicines in Tau-induced toxicity, we modeled tauopathy in the notum of *Drosophila melanogaster.* However, given that the p-Tau protein in *Drosophila* *melanogaster* occurs at extremely low levels and is difficult to detect precisely, we used IMR technology that included the use of antibody-functionalized magnetic nanoparticles and a high-Tc SQUID-based ac magnetosusceptometer. Our IMR data showed that the selected Chinese herbal medicines can significantly reduce p-Tau expression. Using evidence from notal bristle quantification and Western blotting analysis, we confirmed the validity of the IMR data. Technically, SQUID-based IMR technology is suitable for screening Chinese herbal medicines, which is low-cost, low-risk, simple, and highly acceptable. These advantages of IMR technology are critical for the therapeutic evaluation in the tauopathy alleviation of AD *Drosophila* *melanogaster*. Thus, we suggest that IMR can be used as a new tool for measuring tauopathy and the therapeutic evaluation of Chinese herbal medicine in AD *Drosophila melanogaster*. 

## Data Availability

The data are confidential.
